# Alternative Protein Systems: A Bibliometric and Comparative Review of Sustainability, Functionality, and Future Food Systems

**DOI:** 10.3390/foods15183239

**Published:** 2026-09-14

**Authors:** Olena Nifatova, Yuriy Danko, Nataliia Volchenko, Maryna Samilyk, Dmytro Bidiuk, Yana Koplyk

**Affiliations:** 1 The Barcelona Economic Analysis Team, University of Barcelona, 08034 Barcelona, Spain; olena.nifatova@ub.edu; 2 Economics and Management Department, Sumy National Agrarian University, 40021 Sumy, Ukraine; nataliia.volchenko@snau.edu.ua (N.V.); maryna.samilyk@snau.edu.ua (M.S.); d.bidiuk@snau.edu.ua (D.B.); y_koplik@ukr.net (Y.K.)

**Keywords:** alternative proteins, plant-based protein, microbial protein, cultivated meat, insect protein, circular bioeconomy, bibliometric analysis, food systems

## Abstract

The growing demand for sustainable protein sources has accelerated the development of plant-based, microbial, cultivated, and insect proteins. This review combines a systematic analysis of Scopus-indexed publications with bibliometric mapping in Biblioshiny and VOSviewer and a comparative synthesis across environmental, economic, technological, nutritional, and social dimensions. The bibliometric results show a shift from technology-specific studies toward sustainability, circular bioeconomy, life-cycle assessment, consumer acceptance, and protein-transition research. No platform consistently outperforms the others across all dimensions. Plant-based proteins are currently the most technologically mature and economically competitive; microbial proteins offer high resource efficiency and circularity; cultivated meat most closely reproduces conventional meat but remains constrained by cost and energy demand; and insect proteins support nutrient recycling but face persistent acceptance and regulatory barriers. The evidence indicates that future sustainable protein systems will depend on complementary rather than competing production platforms. Integrating these technologies according to their distinct functional and sustainability advantages provides a promising pathway toward resilient and resource-efficient food systems and offers a basis for research priorities, technology development, investment, and policy strategies supporting the global protein transition.

## 1. Introduction

In the context of global population growth, climate change, and increasing pressure on natural resources, identifying sustainable protein production systems has become one of the central challenges for future food security. According to the Food and Agriculture Organization (FAO), the global population is projected to reach approximately 9.3 billion by 2050, while demand for protein is expected to increase by more than 50% compared with 2020 levels [1]. At the same time, conventional livestock production is associated with substantial greenhouse gas emissions, extensive land occupation, and intensive water use [2]. For example, producing 100 g of protein from lamb and beef requires approximately 184.8 and 163.6 m^2^ of agricultural land, respectively [2]. These challenges have intensified interest in alternative protein technologies capable of simultaneously improving resource efficiency, reducing environmental impacts, and strengthening food system resilience.

Alternative protein technologies, including plant-based proteins, microbial proteins, cultivated meat, and insect proteins, have emerged as promising components of the transition toward sustainable and circular agrifood systems. Unlike conventional livestock production, these technologies employ fundamentally different biological pathways and production models, ranging from crop processing and precision fermentation to cellular agriculture and insect bioconversion. Their diversity creates opportunities to reduce dependence on agricultural land, utilize renewable carbon sources and organic side streams, recover nutrients within circular bioeconomy systems, and diversify protein supply chains.

Although numerous review papers have examined individual alternative protein technologies, most focus on a single production platform or emphasize only selected aspects, such as environmental performance, nutritional quality, or consumer acceptance. Comparative studies integrating environmental, economic, technological, nutritional, and social dimensions across the major alternative protein systems remain relatively limited. Consequently, there is still a lack of holistic evidence that would allow a systematic comparison of the strengths, limitations, and future roles of different protein technologies within sustainable food systems.

To address this gap, the present review combines bibliometric analysis with a comprehensive comparative assessment of four major alternative protein platforms: plant-based proteins, microbial proteins, cultivated meat, and insect proteins. Bibliometric mapping is used to identify the evolution of research themes, scientific collaboration patterns, and emerging trends, while the narrative synthesis evaluates each technology across environmental, economic, technological, nutritional, and social dimensions. Based on this integrated approach, the review proposes a comparative sustainability framework that highlights the complementary roles of alternative protein systems within the future protein transition. Rather than identifying a single superior protein technology, this review aims to demonstrate how different alternative protein systems can contribute complementary functions within resilient and sustainable food systems.

## 2. Materials and Methods

### 2.1. Literature Search and Study Selection

This bibliometric analysis was conducted using the Scopus database, selected for its comprehensive coverage of peer-reviewed literature in food science, biotechnology, environmental sciences, agricultural research, and related fields. The search was conducted in May 2026 and covered publications published between 2010 and 2026, reflecting the period during which research on alternative proteins expanded rapidly in response to sustainability and food security challenges.

The search strategy combined terms referring to alternative protein technologies with terms related to sustainability and food-system transformation. The following Boolean search string was applied in Scopus: TITLE-ABS-KEY ((“alternative protein*” OR “plant-based meat” OR “plant protein*” OR mycoprotein OR “single-cell protein” OR “microbial protein” OR “cultivated meat” OR “cell-based meat” OR “cultured meat” OR “insect protein*”) AND (“sustainability” OR “food system*” OR “protein transition” OR “circular bioeconomy”)). Only peer-reviewed journal articles published in English were considered. The search was further restricted to the Scopus subject areas of Agriculture and Biological Sciences, Environmental Science, Social Sciences, Energy, Business, Management, Multidisciplinary, and Economics. The application of the search string and the predefined database filters resulted in 1401 records retrieved from Scopus.

Following the retrieval of the 1401 records, a thematic screening of titles and abstracts was conducted to assess their substantive relevance to the food-system context defined in this study. Records were excluded when alternative proteins were not substantively related to this context. Specifically, studies focused exclusively on non-food biopolymers or biomaterials, medical or pharmaceutical applications of single-cell or microbial proteins, and biofuel or bioenergy production without a substantive food-protein component were excluded. Studies focused exclusively on conventional livestock or aquaculture production, as well as other non-alternative-protein applications, were also excluded when no substantive connection to alternative protein systems was identified. Duplicate records were also removed during the data-cleaning process. After duplicate removal and thematic screening, 1283 publications remained and constituted the final bibliometric corpus used for the analysis.

### 2.2. Bibliometric Analysis

Bibliometric analysis was performed to identify the intellectual structure and thematic evolution of research on alternative proteins. The bibliographic dataset exported from Scopus was analyzed using Biblioshiny (Bibliometrix package for R version 4.3.1) and VOSviewer version 1.6.20.

For VOSviewer analysis, synonymous and closely related keyword expressions were harmonized using a controlled thesaurus. In particular, alternative names referring to the same technological concept were consolidated; for example, “cultivated meat,” “cultured meat,” “cell-based meat,” and “lab-grown meat” were standardized as “cultivated meat.” Abbreviations and spelling variants were standardized where they represented the same concept, while generic indexing terms that did not provide meaningful thematic information were removed. The harmonized author-keyword set was then used for co-occurrence analysis and network visualization in VOSviewer. This procedure reduced lexical fragmentation while preserving the substantive distinctions among the four main alternative protein domains: plant-based protein, microbial protein, cultivated meat, and insect protein.

The bibliometric analysis included publication trends, keyword frequency analysis, thematic evolution, and keyword co-occurrence mapping. These methods enabled the identification of dominant research directions, emerging topics, and relationships among different technological approaches to alternative protein production.

### 2.3. Comparative Analytical Framework

Following the bibliometric analysis, a qualitative comparative framework was developed to synthesize evidence across the four principal alternative protein categories: plant-based proteins, microbial proteins (single-cell proteins), cultivated meat, and insect proteins. Rather than comparing individual experimental results, the framework integrates findings reported across multiple review articles and primary studies.

To ensure consistency of comparison, technologies were evaluated using four sustainability dimensions comprising ten analytical indicators:

(1) Environmental: land use, water use, greenhouse gas emissions, and circularity potential;
(2) Economic: technology readiness level (TRL) and production cost;
(3) Social: consumer acceptance and regulatory maturity;
(4) Nutritional: protein quality and principal industrial applications.

These indicators were selected because they represent the most frequently assessed dimensions identified during the bibliometric analysis and repeatedly discussed throughout the reviewed literature. Together, they provide a multidimensional perspective on the sustainability and industrial potential of alternative protein technologies.

### 2.4. Data Synthesis

The collected evidence was synthesized using a narrative comparative approach. Instead of conducting a quantitative meta-analysis, which was not appropriate due to substantial methodological heterogeneity among life-cycle assessments, techno-economic analyses, consumer surveys, and nutritional studies, the review focused on identifying convergent findings, recurring patterns, and areas of scientific disagreement.

The comparative synthesis summarized the relative strengths and limitations of each protein technology across the selected sustainability dimensions and formed the basis for the integrated assessment presented in Table 9. Particular attention was given to the interactions between environmental performance, technological maturity, nutritional functionality, consumer acceptance, and circular bioeconomy potential, allowing a holistic evaluation of the role of alternative proteins in future sustainable food systems.

## 3. Results (Bibliometric Analysis)

### 3.1. Knowledge Structure and Thematic Evolution of Alternative Protein Research (Bibliometric Mapping and Thematic Structure of Alternative Protein Research)

#### 3.1.1. Global Research Distribution

**Figure 1:**
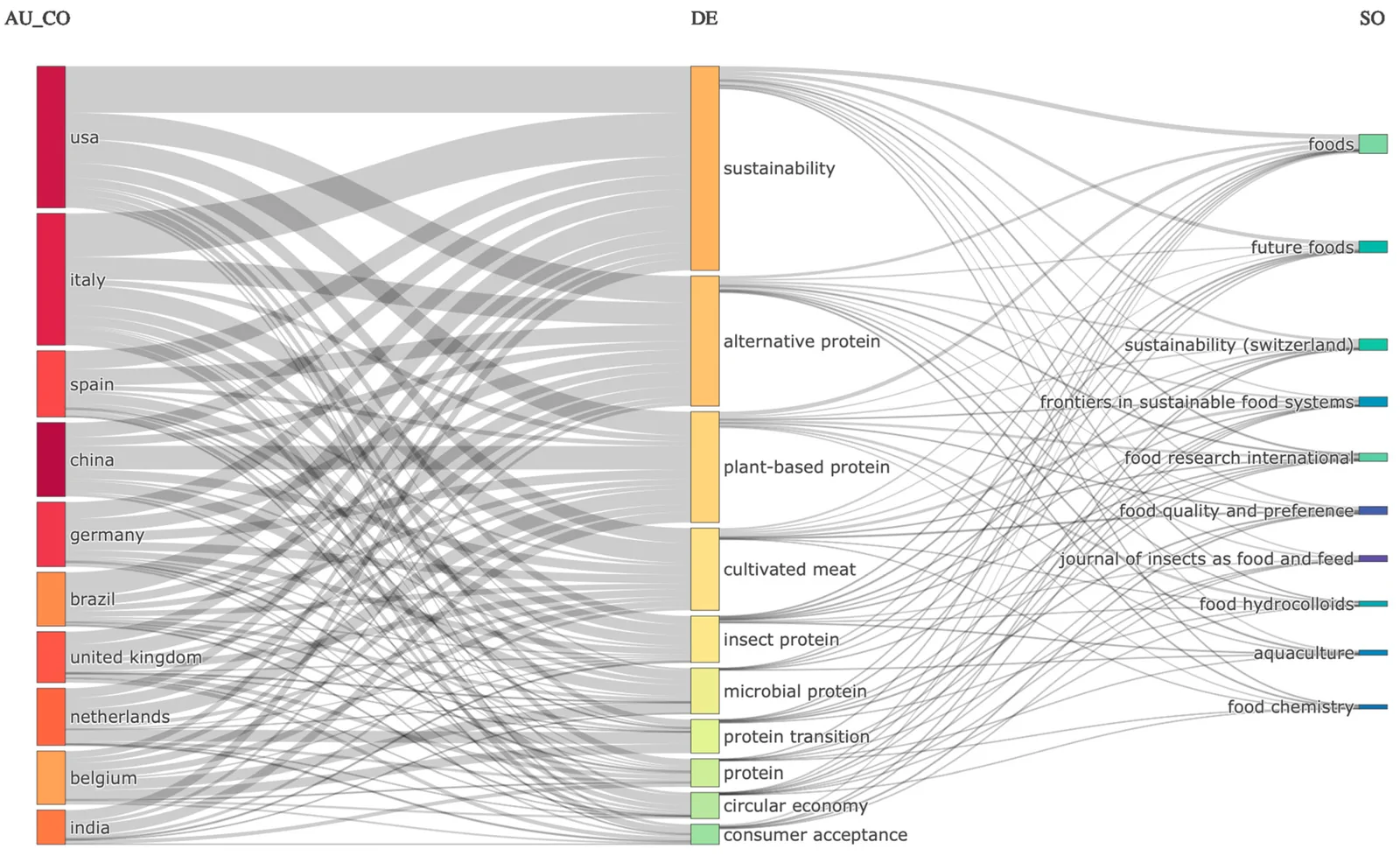
Three-field plot of countries, author keywords, and publication sources in alternative protein research, Biblioshiny.

The global distribution of sustainability-oriented alternative protein research demonstrates a strong concentration of scientific output in developed economies, particularly in the United States, Italy, Spain, China, and Germany (Figure 1). The United States occupies a central position in the knowledge network, showing strong associations with the keywords “sustainability”, “alternative protein”, and “plant-based protein”, which may indicate a leading role in shaping the broader discourse around alternative proteins. European countries, specifically Italy, Spain, the Netherlands, and Belgium, demonstrate equally strong participation in research on the protein transition, consumer acceptance, and circular economy approaches, which may reflect the influence of the European Green Deal and the Farm to Fork policy agenda on scientific priorities. The keyword structure also reveals that sustainability remains the dominant conceptual core of the field, and alternative proteins are increasingly being investigated not as isolated food technologies, but as components of broader sustainability frameworks and food system transformations. At the same time, the emergence of keywords such as “consumer acceptance”, “protein transition”, and “circular economy” indicates a gradual shift in the field towards broader socio-economic perspectives. An analysis of the top 10 journals with the highest concentration of publications shows that the field is positioned at the intersection of food science, sustainability studies, and environmental research. Furthermore, the visible presence of journals such as the Journal of Insects as Food and Feed, Food Hydrocolloids, and Aquaculture reflects the growing specialization of research streams focused on specific protein technologies.

#### 3.1.2. Thematic Evolution over Time (Figure 2)

In the earliest stage (2010–2015), the field is primarily characterized by a narrow focus on the basic nutritional and compositional properties of protein sources, reflecting an exploratory phase of knowledge development. In the subsequent period (2016–2020), the thematic landscape becomes noticeably more diversified. This stage is marked by the emergence of technological and processing-oriented directions, alongside a growing focus on novel protein sources and their functional and production characteristics. The coexistence of agricultural, feed, and food applications suggests a crucial transitional phase in which alternative proteins are being conceptualized within the broader framework of food systems and sustainability. In the most recent period (2021–2026), the thematic structure becomes more consolidated, with a stronger emphasis on edible insects, plant-based protein, cultivated meat, and meat substitutes. The prominence of cultivated meat and meat alternatives may reflect growing attention to solutions to mitigate the environmental impact of conventional animal agriculture.

**Figure 2:**
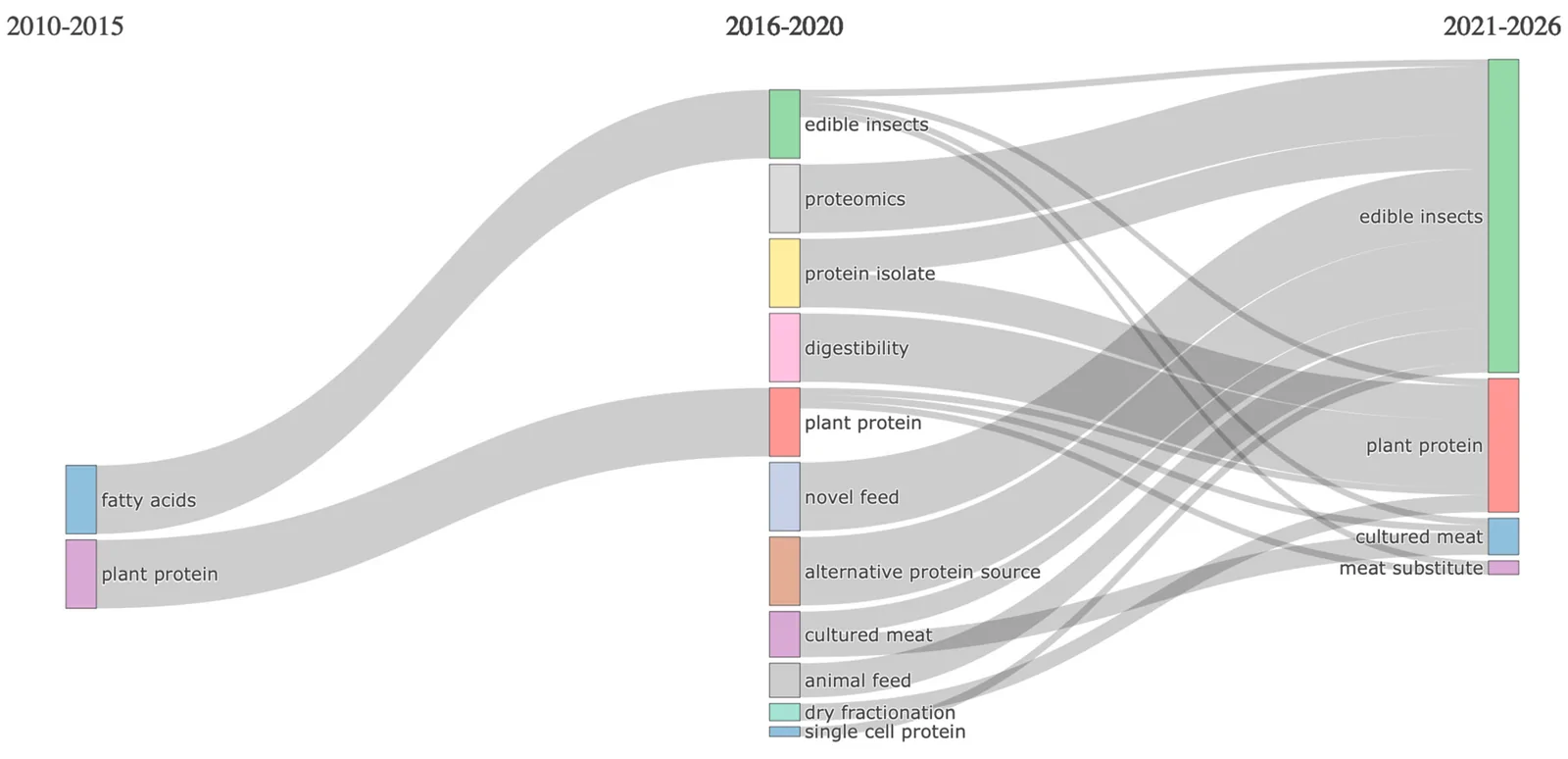
Thematic evolution of alternative protein and sustainability research (2010–2026), Biblioshiny.

#### 3.1.3. Thematic Structure of the Field and Keyword Co-Occurrence Network (VOSviewer)

**Figure 3:**
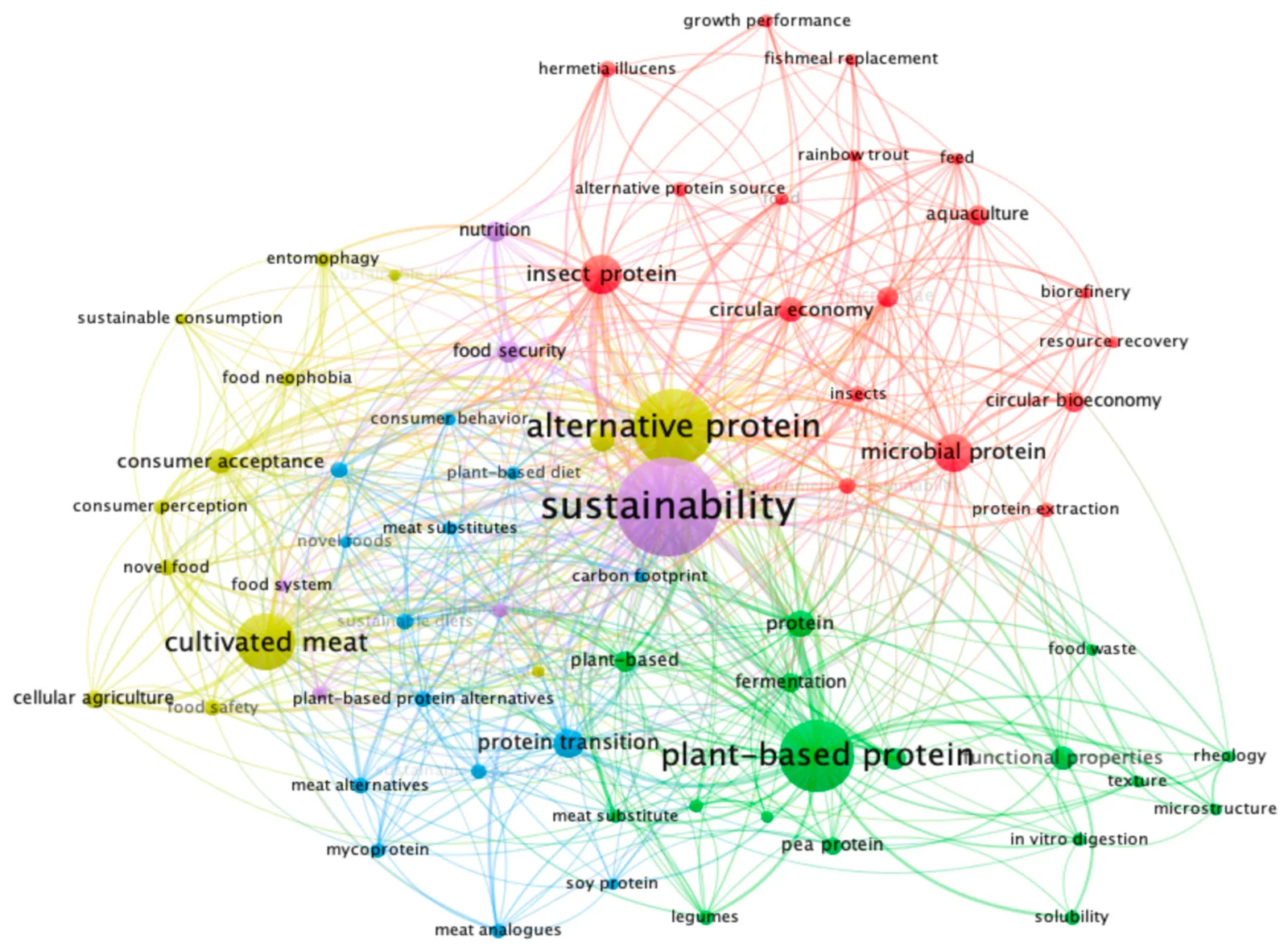
Thematic map of alternative protein research, generated using VOSviewer.

The keyword co-occurrence clustering revealed 5 clusters out of 4075 keywords, based on a minimum occurrence threshold of 10, which yielded 1386 links and distributed the aspects of the thematic structure of alternative protein research into corresponding groups (Figure 3, Table 1).

**Figure 4:**
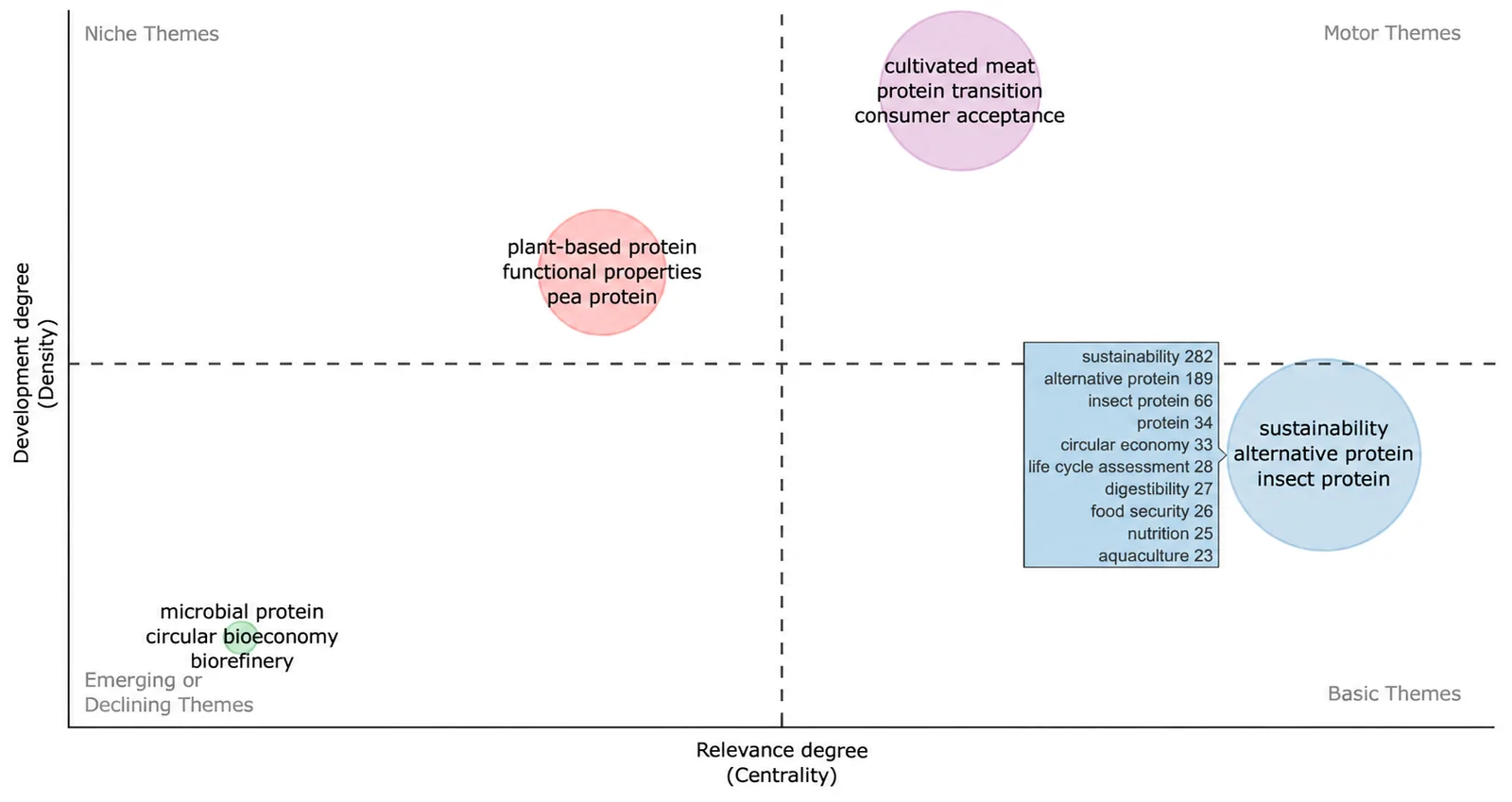
Thematic map of alternative protein research, Biblioshiny.

While co-occurrence clustering demonstrates the structural organization of research streams, the thematic map provides additional insight into their maturity and strategic positioning within the field (Figure 4).

Motor themes, represented by cultivated meat, the protein transition, and consumer acceptance, indicate highly developed and strategically positioned research directions that currently drive the scientific agenda and the integration of technological and behavioral perspectives. Niche themes, including plant-based protein and its associated functional and technological attributes, demonstrate strong internal development but a more specialized scope, reflecting mature yet relatively domain-specific research streams. Basic themes, such as sustainability, alternative protein, and insect protein, occupy a structurally fundamental position in this field, serving as broadly connected conceptual markers despite varying levels of thematic development. In contrast, microbial protein—in conjunction with the circular bioeconomy and biorefineries—emerges as a new or less consolidated theme, suggesting either early-stage scientific exploration or a more peripheral role in the current configuration of the research landscape.

## 4. Overview of Alternative Protein Production Systems and Sources

### 4.1. Plant-Based Protein

Plant-based protein is the most widespread alternative to traditional animal protein sources, utilized in the food industry as a plant-based meat substitute. These proteins can be derived from a diverse range of plant raw materials. The most common raw materials include legumes, cereals, oil crops, nuts, and seeds, which are processed into protein ingredients such as concentrates and isolates. Plant proteins serve as the primary structural components for creating plant-based meat analogues, given their ability to mimic the fibrous texture, flavor, and appearance of animal-derived products [3]. The market dominance of plant proteins is driven by a combination of the economic affordability of raw materials, technological maturity, and high consumer acceptance [4,5,6]. Compared to conventional animal agriculture, the production of these proteins is significantly more environmentally sustainable, requiring fewer land and water resources while generating lower greenhouse gas emissions [7]. From a nutritional perspective, plant-based protein sources generally contain no dietary cholesterol and tend to have lower levels of saturated fat than animal-derived protein sources [8].

Despite these significant advantages, the utilization of plant proteins presents certain limitations related to their nutritional value and organoleptic characteristics. Many plant sources possess an “incomplete” amino acid profile; for instance, legumes frequently lack sulfur-containing amino acids (methionine), while cereals are limited in lysine [9]. Furthermore, the presence of antinutrients can hinder mineral absorption, while specific undesirable off-flavors, such as grassy or “beany” notes, necessitate technological masking [4].

#### 4.1.1. Sources of Plant Proteins

The selection of a specific protein source is based on its techno-functional properties, such as gelation, emulsification, and water- and fat-binding capacities, which are critical for mimicking the fibrous structure and juiciness of meat. In addition to traditional crops such as soy, pea, and wheat gluten [5], researchers are exploring the potential of novel and underutilized sources—such as mung bean, lupin [10], pumpkin seed, moringa, and hemp [11,12]—which facilitate the balancing of the amino acid profile and the overcoming of certain organoleptic limitations. Table 2 provides a consolidated comparative overview of the primary plant-based protein sources based on their protein content, functional properties, application areas, and key limitations.

#### 4.1.2. Technologies for Processing and Structuring Plant-Based Proteins

The technological cycle of producing plant-based meat analogues is a multi-stage process aimed at overcoming the natural differences between the globular structure of plant proteins and the anisotropic architecture of animal muscle tissue. Overall, the technological process of forming plant-based meat substitutes can be divided into two main stages:

Protein extraction and modification. This stage involves the extraction of proteins from the plant matrix to obtain concentrates (50–70% protein) or isolates (>90%), as well as their subsequent physical, chemical, or biological modification. These modifications are designed to improve techno-functional properties, such as solubility and gelation, and to eliminate off-flavors.

Protein structuring. This stage focuses on the creation of textured structures. Ulhas et al. [9] classify structuring technologies into two approaches: bottom-up and top-down approaches. The bottom-up approach involves the formation of individual structural elements at the nanoscale and their subsequent assembly into a cohesive product to replicate the hierarchical architecture of meat. The top-down approach involves mimicking the fibrous texture on a larger scale through thermal and mechanical processing of biopolymer mixtures to obtain anisotropic structures. Each of the structuring technologies provides a different level of replication of the fibrous meat structure and is characterized by specific advantages and limitations in terms of final product quality, production efficiency, and commercial readiness. A comparative characterization of the primary plant-based protein structuring technologies is presented in Table 3.

### 4.2. Microbial Protein

Microbial proteins are commonly discussed within the broader concept of single-cell protein (SCP), although contemporary microbial protein systems increasingly include more diverse biotechnological production pathways. The technology for producing microbial proteins involves the cultivation of microorganisms such as bacteria, filamentous fungi, yeasts, and microalgae. The basis for such cultivation is the use of various substrates. The first generation of microbial proteins involves the use of sugars as substrates [18], while the second generation is based on non-food raw materials, such as agricultural residues [19]. Additionally, the second generation of microbial proteins offers new solutions related to the genetic modification of microorganisms and the improvement of bioreactor configurations, which enhance protein biomass production [20]. Recent studies also propose technological solutions where carbon dioxide (CO_2_) is used as a substrate through a gas fermentation process [21]. This technology has gained popularity due to its dual environmental benefits: on one hand, using waste gases as a substrate helps reduce CO_2_ emissions, thereby mitigating the impact on climate change; on the other hand, the technology allows for the production of high-quality protein that contains sufficient amino acids and has high digestibility [22], making it suitable as an ingredient in animal feed and even in human food products. Microbial proteins are increasingly considered a promising strategy for addressing global food security challenges. Compared to traditional livestock farming, microbial biomass production has advantages such as significant resource savings due to minimal use of agricultural land and water, as well as the rapid growth rate of microorganisms (microbial biomass can be produced within weeks, whereas conventional livestock production typically requires substantially longer production cycles) [23]. Moreover, unlike traditional livestock farming, the production of such protein is not dependent on climate conditions, making it more resilient in this regard. However, it should be noted that the main criteria for optimizing the production process and economic efficiency of the technology are the choice of substrate and microbial platform for protein biomass production.

#### 4.2.1. Selection of Substrates for Growing Microbial Protein

In general, Raziq et al. [24] classify potential substrates for SCP production into four groups: energy-intensive substrates, biofeedstocks derived from biological raw materials, biowastes originating from agricultural and other organic residues, and carbon dioxide (CO_2_)-based substrates. In this classification, biofeedstocks refer to biological raw materials that can be used as inputs for microbial cultivation, whereas biowastes are residual materials generated from agricultural, food-processing, and other production activities. The choice of substrates for growing single-cell proteins depends on factors such as waste availability, logistical costs and transportation conditions, the need for pre-treatment and its cost, and the efficiency of the bioprocess, i.e., the protein content of the resulting biomass [25].

Current research increasingly emphasizes the use of low-cost and waste-derived substrates for microbial protein production within circular bioeconomy systems. Agro-industrial residues, municipal waste streams, and industrial by-products are considered particularly promising due to their wide availability and potential for resource recovery [26]. Among the most commonly investigated substrates are lignocellulosic biomass, food processing residues, grape pomace, municipal wastewater, whey from cheese production, raw glycerin from biodiesel production, and wastewater from the paper and pulp industry [23,26]. These substrates not only reduce environmental burdens associated with waste disposal but also provide nutrient-rich media for microbial growth and protein biomass production.

Special attention has recently been devoted to methane- and CO_2_-based substrates due to their potential contribution to climate change mitigation. Methanotrophic bacteria capable of converting methane into protein biomass have demonstrated promising results, particularly in aquaculture applications where microbial proteins can partially replace fishmeal without negatively affecting fish growth or health [27]. At the same time, economically viable substrates increasingly include inexpensive agro-industrial by-products such as molasses and corn steep liquor, which supply essential nutrients for microbial growth while reducing overall production costs [28]. Overall, substrate selection plays a central role in determining the environmental performance, economic feasibility, and scalability of microbial protein production systems.

#### 4.2.2. Selection of Microbial Platforms for Microbial Protein Production

Beyond substrate selection, the efficiency and environmental performance of microbial protein production are also strongly influenced by the choice of microbial platform [18]. Filamentous fungi, microalgae, and bacteria represent the most commonly used microbial platforms. Yeasts and mixed microbial cultures are also considered as potential platforms [23].

Filamentous *fungi* are among the most widely used microbial platforms for microbial protein production due to their high protein yield, favorable amino acid composition, and ability to utilize diverse low-cost substrates [29]. Their cultivation is considered technologically attractive because of relatively simple fermentation requirements and flexibility in substrate selection, including lignocellulosic biomass and agro-industrial residues. A prominent example is *Fusarium venenatum*, which has been commercially used for the production of the meat alternative Quorn™ since 1985 [30]. In addition to high protein content, fungal biomass contains vitamins, lipids, and bioactive compounds, making it suitable for food, aquaculture, poultry, and livestock applications. However, certain filamentous fungi may produce mycotoxins or trigger allergic reactions, creating challenges for large-scale commercialization and food safety management.

Among microbial platforms, *microalgae* have attracted increasing attention due to their rapid biomass accumulation and ability to utilize waste-derived substrates. Examples of microalgae investigated as protein sources include *Chlorella vulgaris*, *Scenedesmus* spp., *Nannochloropsis* spp., and *Arthrospira (Spirulina) platensis*, which are characterized by relatively high protein contents and potential applications in food and feed systems [31]. Microalgae can also be cultivated using agri-industrial wastewaters, enabling the simultaneous recovery of carbon, nitrogen, and phosphorus into microbial biomass; however, the resulting biomass may present challenges related to product quality and algal harvesting [32]. A further limitation is the need for downstream processing to disrupt the rigid cell walls of many microalgae and improve protein extraction and digestibility, although their rapid growth and potential use of low-cost substrates make them promising platforms for sustainable microbial protein production [31].

*Bacteria* are another alternative for growing microbial proteins. Their short generation time and even faster growth rates compared to fungi and algae (bacterial growth rate ranges from 0.28 µm to 0.65 µm per hour, while algae and fungi grow at rates from 0.16 µm to 0.39 µm per hour [18] create significant economic advantages. The genus *Methylotrophus* is the most commonly used for commercial single-cell protein production. This type of bacterium uses methane as a substrate by assimilating nitrogen, which leads to protein production. Another type of bacterium is *Rhodobacter capsulatus*—a phototrophic bacterium that uses food waste as a substrate. In this context, organic waste refers to biodegradable materials of biological origin, such as food and agricultural residues, whereas chemical waste refers to waste streams or chemical substrates containing compounds such as methanol or ethanol that can serve as carbon and energy sources for microorganisms. However, the presence of toxins due to high levels of nucleic acids and low cell density, which complicates the harvesting process, creates limitations for its use. These limitations can be addressed through optimization of fermentation conditions, including bioreactor operation, substrate selection, and process parameters, as well as through downstream treatments to reduce the nucleic acid content of the resulting biomass [33].

*Yeasts* are also widely used for microbial proteins production, mainly due to their high protein content in the final product (45–55% dry weight), the presence of B vitamins, and their versatility in substrate usage (whey waste, agricultural residues, cellulose hydrolysates, etc.). They easily adapt to acidic pH and are generally recognized as safe due to their long-standing use in traditional fermentation processes [34]. *Yarrowia lipolytica* is noted for its high lipid and protein content, while *Candida utilis* is rich in lysine and vitamins. The main limitations are the risk of pathogenicity of certain species (e.g., *C. krusei*) and the need to monitor the process to prevent contamination.

#### 4.2.3. Integration of Substrates, Microbial Platforms, and Fermentation Processes

The production of microbial proteins requires the integration of three interdependent components: substrate selection, microbial platform choice, and bioprocess configuration. The efficiency, environmental performance, and economic feasibility of microbial protein production largely depend on the interactions among these elements. To systematize the reviewed approaches, Table 4 summarizes the comparative characteristics of major microbial platforms.

**Figure 5:**
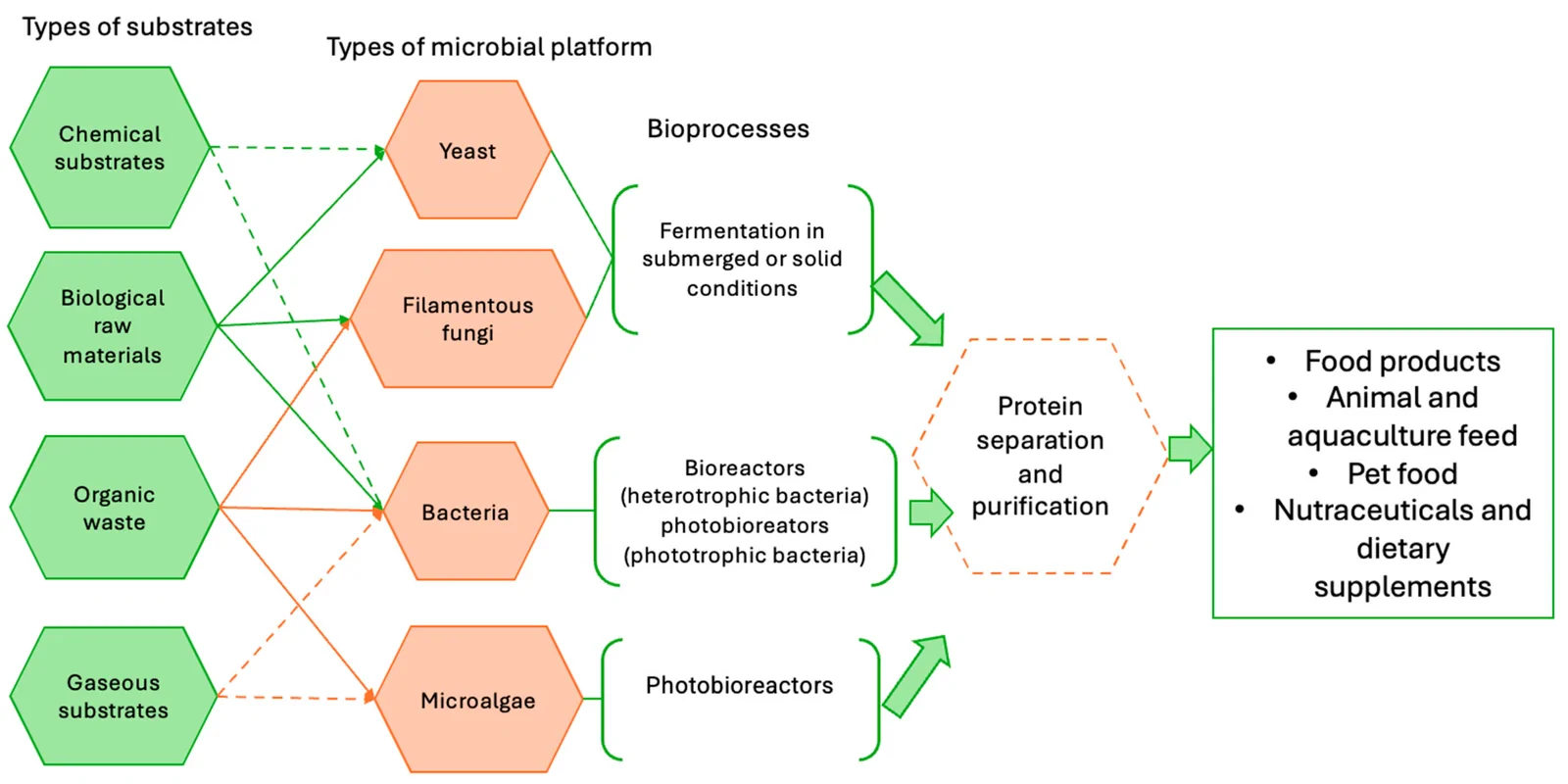
Integrated framework of substrate utilization, microbial platforms, and fermentation processes for microbial protein production.

Figure 5 summarizes the relationship between the four primary stages of microbial protein production: feedstock (substrate) selection, microbial platform choice, the type of bioprocesses (fermentation) applied, and the final stage of product downstream processing.

### 4.3. Cultivated Meat (Cell-Based Meat)

Cultivated meat (also known as cell-based meat or lab-grown meat) represents a paradigm shift in food production systems, transitioning from conventional livestock farming to a cellular biomanufacturing system [35]. In contrast to conventional livestock production, this technology relies on the controlled in vitro proliferation and differentiation of animal-derived cells within specialized bioreactors [36]. This approach allows the final product to be viewed not merely as a discrete “ingredient,” but as a complex biological structure that aims to replicate the architecture of muscle, adipose, and connective tissues under laboratory conditions [37].

The technological cycle of cultivated meat production relies on two core blocks. The first is the selection of cell sources, which constitutes the biological foundation of the process and involves the isolation of high-quality stem cells or progenitors, such as muscle satellite cells, mesenchymal stem cells, or pluripotent lines, from livestock or aquaculture organisms [35,38]. The second block is tissue engineering, which drives the biofabrication transformation through cell mass expansion and its subsequent structuring using scaffolds, bioinks utilized in bioprinting approaches, or cell sheet technologies to form a functional and organoleptic equivalent of conventional meat [37].

The key distinction between cultivated meat and plant-based or microbial proteins lies in its biological nature and the end result. While plant-based analogues utilize plant-derived proteins and other botanical ingredients to reproduce the structural and sensory characteristics of meat, and microbial proteins are obtained through the fermentation of microorganisms, such as fungi, yeasts, or algae, cultivated meat consists of animal-derived cells that form tissue-like structures. This allows for the creation of a product that is comparable to conventional meat in terms of structure and nutritional profile, thereby overcoming the sensory limitations inherent to other alternative proteins [36].

#### 4.3.1. Cell Sources for Cultivated Meat Production

The choice of cell source is a fundamental biological decision that determines the efficiency of the entire cultivated meat biomanufacturing system. The characteristics of the selected cell line directly influence the rate of tissue formation, industrial scalability, and final organoleptic properties of the product. Modern scientific literature distinguishes two primary categories, including adult stem cells, specifically muscle satellite cells (MuSCs) and mesenchymal stem cells (MSCs), as well as pluripotent cell platforms, namely embryonic stem cells (ESCs) and induced pluripotent stem cells (iPSCs) [36,39]. The primary technological trade-off lies in choosing between the high specialization and genomic stability of MuSCs, which possess a naturally limited division cycle, and the theoretically unlimited self-renewal capacity of ESCs and iPSCs. Although the latter provide the necessary foundation for mass production, they require more complex differentiation protocols and strict regulatory oversight due to ethical concerns and risks of genetic instability [40].

A detailed comparative characterization of the primary cell sources and their operational parameters is presented in Table 5. The selected cell sources represent the most widely discussed and functionally relevant platforms in cultivated meat research.

#### 4.3.2. Integrated Cultivation Systems and Bioprocess Architecture

Cultivated meat production relies on an integrated bioprocessing pipeline that combines sequential and interdependent technological modules, ranging from cell expansion to final tissue structuring [38]. Rather than operating as isolated techniques, these components function as a coordinated system that replicates key aspects of animal tissue formation under controlled in vitro conditions. The process begins with the expansion of selected cell populations, followed by cultivation in bioreactor systems that regulate physicochemical parameters such as oxygen transfer, nutrient supply, and shear stress. Structural development is then guided through scaffold-based platforms and biofabrication strategies that enable three-dimensional tissue organization. Finally, process intensification approaches are employed to improve efficiency, reduce production costs, and enhance scalability [35]. This system-level perspective conceptualizes cultivated meat production as an integrated manufacturing pipeline rather than a collection of discrete biotechnological methods (Table 6). Bioreactor systems are the most technologically mature. Concurrently, process intensification systems significantly affect the economic feasibility of production, determining the potential to achieve price parity with conventional meat. Consequently, the primary challenge for the commercialization of cultivated meat is not an isolated technology, but rather the necessity for the harmonized development of all components within the production chain.

### 4.4. Insect Protein

Insects have emerged as a pivotal alternative protein source within modern circular bioeconomy frameworks [46]. Unlike traditional livestock, insect biomanufacturing systems offer a sustainable pathway for protein production by transforming low-value organic side-streams and bio-waste into high-quality biomass. This capacity for waste valorization, combined with exceptional resource efficiency, evidenced by significantly lower land, water, and energy requirements, positions insects as a critical solution for global food security [47]. Characterized by rapid growth rates and high feed-to-food conversion efficiencies, insects generally provide high-quality protein containing all essential amino acids, although the amino acid profile varies among species, while offering environmental advantages superior to conventional animal agriculture. Within the broader landscape of alternative proteins, insects represent a unique animal-based system that aligns ecological preservation with the production of nutrient-dense food and feed.

#### 4.4.1. Insect Species Used for Protein Production

Numerous insect species are currently under investigation as viable platforms for sustainable protein production. However, they differ considerably in protein content, production characteristics, industrial maturity, and end-use applications. Species selection is further influenced by regulatory approval, production scalability, substrate utilization, and food or feed safety requirements. These differences determine the suitability of individual species for specific market segments, including human food, animal feed (including aquaculture), pet food, and waste valorization. Table 7 summarizes the comparative characteristics of the principal insect species currently used for protein production.

#### 4.4.2. Insect Protein Production Systems and Bioconversion Pathways

Beyond species-specific biological traits, the industrial viability of insect proteins is determined by integrated bioprocess architectures structured into distinct bioconversion pathways. These pathways are defined by substrate characteristics, rearing environments, and the intensity of downstream processing. Current research identifies three primary production models: (1) waste-derived bioconversion systems, which utilize manure and municipal organic waste to generate crude biomass and bio-fertilizers [54]; (2) agro-industrial by-product systems, leveraging residues like wheat bran and food processing waste for standardized food and feed-grade flours; and (3) controlled optimized rearing systems, which employ high-intensity technologies, such as enzymatic hydrolysis and ultrasound-assisted extraction, to produce high-value protein isolates and bioactive peptides [55]. These systems vary significantly in technological complexity and industrial scalability, targeting diverse applications ranging from bulk animal feed to specialized functional ingredients. A detailed framework of these system-level bioprocess architectures is provided in Table 8.

**Table 8:** Insect-based protein production systems: substrate pathways, outputs, and scale-up constraints.

Production System	Rearing Substrate Base	Output Product Streams	Key Scale-up Constraints	References
Waste-derived bioconversion systems	Animal manure (poultry, pig), catering waste, municipal organic waste, sewage sludge	Whole dried insect biomass; crude insect meal; organic fertilizer (frass)	Pathogen and heavy metal accumulation; high labor intensity without automation; safety control of waste-derived inputs	van Huis [47]; Pan et al. [48]
Agro-industrial by-product systems	Wheat bran, brewery by-products, distillery residues, coffee silverskin, fruit/vegetable processing waste	Defatted insect meal; protein concentrates; standardized insect flour	High energy demand in drying and fractionation; variability of substrate composition; cost of advanced extraction technologies	Queiroz et al. [55]; Pan et al. [48]; Morales-Ramos et al. [56]
Controlled optimized rearing systems (feed-grade/R&D systems)	Standardized reference diets; formulated plant-based feeds; nutrient-controlled substrates	Protein isolates; hydrolysates; bioactive peptides	High production cost; enzyme cost and process complexity; regulatory constraints (novel food approval); scalability limitations for high-value fractions	Matos et al. [54]; Ma et al. [57]; Morales-Ramos et al. [56]

## 5. Comparative Sustainability Assessment of Alternative Protein Production Systems

The transition toward a resilient global food system necessitates a radical rethinking of protein production to mitigate the environmental and ethical challenges associated with intensive livestock farming [58,59]. While the preceding sections provided a detailed examination of plant-based, microbial, cultivated, and insect-based systems as isolated technological modules, their viable integration into the human diet requires a comprehensive multidimensional comparison. A systemic perspective is essential because sustainability cannot be reduced to a single metric; relying solely on GHG emissions, for instance, may mask critical trade-offs in water scarcity, land requirements, or nutritional bioaccessibility [60].

Table 9 synthesizes the evidence base across four primary sustainability dimensions: environmental, economic, social, and nutritional. Each category is evaluated through harmonized and comparable indicators, including land use, water use, GHG emissions, and circularity potential for ecological assessment. Economic and technological feasibility are represented by technology readiness levels (TRL) and production costs, reflecting the maturity of the manufacturing pipelines. The social dimension is addressed through the lens of consumer acceptance, accounting for familiarity and food neophobia, and regulatory maturity, particularly within Novel Food frameworks. Finally, the nutritional functionality dimension compares protein quality and major industrial applications to determine how each system fulfills specific dietary roles. This comparison highlights the principal strengths, limitations, and strategic roles of each alternative protein technology, providing the analytical foundation for the subsequent discussion on their complementary implementation in future food systems.

**Table 9:** Comparative synthesis of alternative protein systems across environmental, economic, nutritional and social dimensions.

Sustainability Dimension	Indicator	Plant-Based Protein	Microbial Protein (SCP)	Cultivated Meat	Insect Protein	Comparative Interpretation	Key References
Environmental	Land use	**Low** requires agricultural land but substantially less than livestock	**Very low** decoupled from arable land; produced in bioreactors with vertical scalability	**Very low** potential for up to 99% reduction in land use compared to conventional beef	**Low** high-density vertical rearing systems minimize spatial footprint.	Cell-based and microbial systems show the greatest land use efficiency	Mazac et al. [60]; Ojha et al. [61]; Hawkey et al. [62]; Sinke et al. [63]; Wali et al. [64]; Rubio et al. [65]
	Water use	**Low** lower than livestock, although irrigation may be substantial for some crops	**Very low** closed fermentation systems enable efficient water recycling	**Medium-Low** culture media preparation increases water demand	**Low** insects require little water due to poikilothermic metabolism	Microbial systems demonstrate the highest water-use efficiency.	Mazac et al. [60]; Hawkey et al. [62]; Gundupalli et al. [66]; Bajić et al. [23]
	GHG emissions	**Low** lowest among current commercial alternatives	**Low** depends largely on energy source	**Medium-Low** environmental advantage depends on low-carbon electricity	**Low** substantially lower methane emissions than livestock	Plant-based proteins currently exhibit the lowest carbon footprint, whereas cultivated meat remains energy-dependent.	Mazac et al. [60]; Sinke et al. [63]; Gundupalli et al. [66]; Lynch and Pierrehumbert [67]
	Circularity potential	**Medium** utilizes agricultural by-products and oilseed cakes	**High** capable of converting CO_2_, methane, industrial gases and waste streams	**Low** relies on high-purity culture media with limited recycling	**Very high** Very high; converts food waste and manure into protein and fertilizer	Insects and microbial proteins provide the strongest contribution to circular bioeconomy.	Gundupalli et al. [66]; Koukoumaki et al. [68]; Pereira et al. [26]; Bajić et al. [23]
Economic	Technology readiness (TRL)	**TRL 9** fully commercialized	**TRL 7–9** fungal SCP is commercial, bacterial systems are emerging	**TRL 4–6** pilot-scale commercialization	**TRL 8–9** commercial in feed, expanding in food	Plant-based proteins are the most mature technology, followed by insect and microbial proteins.	Hamlin et al. [69]; Akinmeye et al. [70]; Pereira et al. [26]; Sinke et al. [63]
	Production cost	**Low** benefits from existing agricultural and processing infrastructure	**Medium** capital-intensive fermentation and downstream processing	**High** expensive media, growth factors and bioreactors	**Medium-Low** costs associated with labor, drying, and energy; dropping with automation.	Cultivated meat remains economically constrained, whereas plant proteins are currently the most cost-competitive.	Hamlin et al. [69]; Akinmeye et al. [70]; Pereira et al. [26]; Bajić et al. [23]; Sinke et al. [63]
Social	Consumer acceptance	**High** perceived as familiar and natural	**Medium** generally acceptable but “microbial” origin may reduce willingness	**Low** concerns regarding unnaturalness and biotechnology	**Low** in Western countries; strongly affected by food neophobia	Consumer perception remains the principal barrier for insect protein and cultivated meat	Giacalone and Jaeger [58]; Koukoumaki et al. [68]; Medeiros et al. [71]; Akinmeye et al. [70]
	Regulatory maturity	**Established** Standard food regulations apply; labeling is the primary point of debate.	**Mature** Mature Novel Food framework and long history of fermentation products	**Early** Emerging regulatory frameworks; approved only in a few countries	**Emerging** Rapid authorization of specific species (e.g., T. molitor) in the EU.	Plant-based proteins currently benefit from the most predictable regulatory environment	Gundupalli et al. [66]; Akinmeye et al. [70]; Grasso et al. [6]; Sinke et al. [63]; Mazac et al. [60]
Nutritional	Protein quality	**Medium** Good but sometimes limited by essential amino acid profile	**High** High-quality protein with balanced amino acid composition	**Very high** Equivalent to conventional animal protein	**High** High biological value and rich in essential amino acids	Cultivated meat, microbial proteins and insects generally provide more complete amino acid profiles than plant proteins	Bajić et al. [23]; Gundupalli et al. [66]; Pereira et al. [26]; Medeiros et al. [71]; Koukoumaki et al. [68]
	Main industrial applications	Meat analogues, dairy alternatives, beverages, protein ingredients	Food ingredients, feed, protein concentrates, fermentation-derived products	Whole-cut meat analogues and premium meat products	Animal feed, protein flour, functional ingredients	Each protein category occupies a distinct technological and market niche rather than directly replacing the others.	Pereira et al. [26]; Sinke et al. [63]; Akinmeye et al. [70]

Plant-based proteins demonstrate the highest technological maturity and the strongest integration into existing food markets among the four categories. Their established processing infrastructure, broad raw-material base, and applicability to meat and dairy alternatives support relatively efficient commercial production. However, their ability to reproduce the structural and sensory characteristics of whole-muscle meat remains limited, particularly with respect to tenderness and juiciness [5].

Microbial proteins demonstrate high resource-use efficiency and substantial potential for production using non-arable resources and alternative carbon and nutrient sources [21,66]. Their production can be integrated with agro-industrial residues and waste streams, creating opportunities for resource recovery and biomass generation [23,32]. However, high nucleic acid content in some microbial biomass, downstream processing requirements, fermentation infrastructure, and consumer unfamiliarity remain important constraints.

Cultivated meat offers the potential to reduce dependence on land-intensive livestock farming while maintaining the biological characteristics of animal meat. Its principal strength is the potential to deliver a genuine sensory experience and nutritional profile similar to conventional meat. Major limitations include its high energy requirements and current reliance on expensive, food-grade culture media and growth factors. The technology is particularly relevant to applications requiring high structural and sensory similarity to conventional meat, where plant-based alternatives fail to achieve parity. However, significant technological bottlenecks in industrial-scale bioreactor engineering and societal concerns regarding the «unnaturalness» of lab-grown products remain the primary hurdles to mass-market entry [72].

Insect proteins combine high feed-conversion efficiency with opportunities to valorize organic side streams and recover nutrients within circular production systems [61]. Their applications extend from animal feed and aquaculture to food ingredients and other higher-value products. Wider adoption is nevertheless constrained by food-safety requirements, variability in substrate composition, regulatory considerations, processing requirements, and limited consumer acceptance in many Western markets [71].

## 6. Discussion

The comparative synthesis indicates that the sustainability performance of alternative protein systems is inherently multidimensional and cannot be assessed using a single criterion. None of the four protein categories demonstrates a consistent advantage across all evaluated dimensions. Instead, the reviewed literature highlights complementary strengths and limitations, suggesting that their relative performance depends on the specific environmental, technological, economic, nutritional, or social perspective considered. These differences largely reflect the fundamentally distinct production pathways underlying each protein category, together with variations in technological maturity, energy requirements, consumer acceptance, and regulatory development.

### 6.1. Environmental Impact and Circularity Potential

There is broad agreement across multiple studies that plant-based and insect-based systems offer the most immediate reductions in land and water use. Mazac et al. [60] reported that incorporating novel foods into meals can reduce global warming potential and land use by over 80%. However, a significant area of disagreement exists regarding the long-term climate impact of cultivated meat. While several life-cycle assessments report substantial environmental benefits of cultivated meat, Lynch and Pierrehumbert [67] cautioned that these advantages may not be fully realized under carbon-intensive energy systems. They argued that substituting short-lived methane emissions from livestock with persistent fossil-fuel-derived CO_2_ emissions could lead to less favorable long-term climate outcomes than commonly assumed. In contrast, Sinke et al. [63] suggested that these conclusions are highly sensitive to assumptions regarding future energy systems. Their life-cycle assessment indicates that the environmental performance of cultivated meat improves substantially under scenarios involving the progressive decarbonization of electricity generation.

Several recent reviews identify substrate selection as one of the key determinants of the environmental performance of insect and microbial protein production systems. Smetana et al. [73] demonstrated that the sustainability advantages of insect protein depend strongly on the rearing substrate: insects cultivated on high-quality cereal feed exhibited considerably higher environmental impacts, whereas the utilization of manure or organic side-streams substantially improved overall environmental performance. Similar conclusions have been reported for microbial proteins. Gundupalli et al. [66] and Koukoumaki et al. [68] emphasized that single-cell proteins can utilize CO_2_, methane, and other industrial waste gases as carbon sources, thereby reducing dependence on arable land and strengthening their role within circular bioeconomy systems. Together, these findings suggest that both insect and microbial proteins are determined not only by the biological characteristics of the production organism but also by the integration of these technologies into circular resource management systems.

### 6.2. Nutritional Functionality and Consumer Acceptance

While all four alternative protein systems provide nutritionally valuable protein, they fulfill different functional roles within the food system. Plant-based proteins are currently the most established ingredients for meat analogues, although Sha and Xiong [5] noted that they still struggle to reproduce the anisotropic fibrous structure of whole-muscle tissue, limiting their application mainly to restructured products. In contrast, cultivated meat is the only technology capable of reproducing the cellular architecture and nutritional profile of conventional meat. Insect and microbial proteins primarily function as high-quality protein ingredients rather than direct meat replacements, offering balanced amino acid profiles and broad applications in both human food and animal feed.

Despite these nutritional advantages, consumer acceptance remains highly uneven across alternative protein categories. Hamlin et al. [69] observed that cultivated meat frequently evokes perceptions of unnaturalness despite its potential environmental and ethical benefits. Similarly, Giacalone and Jaeger [58] described cultured meat as belonging to a group of food technologies that consumers adopt more slowly than innovations perceived as less disruptive. Experimental evidence confirms that perceived naturalness and disgust are significant determinants of cultivated meat acceptance, while the multinational study by Siegrist and Hartmann [74] conducted across ten countries demonstrated substantial cross-cultural differences and identified perceived naturalness, disgust, trust, and food neophobia as important predictors of acceptance. Consumer acceptance of insect protein is constrained by food neophobia and disgust. In a German survey of 516 consumers, food disgust was the strongest predictor of willingness to consume insect-based foods, followed by previous insect consumption, food neophobia, and food technology neophobia [75]. Other empirical studies further show that familiarity and product presentation influence acceptance: Dutch consumers evaluated insect products more positively when they were presented in less recognizable forms, while food neophobia remained an important individual-level barrier [76]. Microbial proteins benefit from the long history of fermentation-based foods, although the microbial origin itself may still reduce willingness to consume among some consumers. This pattern is also evident for mycoprotein: a European consumer study found that 56% of participants were not directly familiar with fungal proteins, while food technology neophobia was associated with purchase intentions and familiarity with fungal protein was positively related to acceptance [77].

Taken together, these empirical findings indicate that consumer responses to alternative proteins are shaped not only by nutritional or technological characteristics but also by psychological factors, prior experience, familiarity, perceived naturalness, and culturally embedded food preferences.

### 6.3. Policy Implications and Future Protein Systems

The findings of this review indicate that alternative protein technologies should be considered complementary components of resilient food systems rather than direct competitors. This interpretation is consistent with Sinke et al. [63], who argued that the greatest environmental benefits can be achieved through the partial substitution of high-impact animal proteins with a diversified portfolio of alternative protein sources. Within such a portfolio, plant-based proteins provide the most cost-effective and technologically mature solution, cultivated meat addresses demand for products with sensory characteristics similar to conventional meat, while microbial and insect proteins contribute primarily through resource efficiency, waste valorization, and circular bioeconomy applications. These complementary strengths suggest that future protein systems should capitalize on the comparative advantages of each technology instead of pursuing a single universal replacement.

These findings also have direct implications for food-system policy, as the different technological maturity, resource-use profiles, and market barriers identified across the four protein categories call for differentiated policy support rather than a uniform approach to alternative proteins. In the European Union, this direction is consistent with the Farm to Fork Strategy, which promotes the transition toward food systems that combine environmental performance, food security, affordability, and innovation, and with the Food 2030 research and innovation framework, which explicitly identifies alternative proteins for dietary shift, circularity and resource efficiency as priorities for food-system transformation [78]. From this perspective, plant-based proteins can benefit from policies supporting domestic protein production, sustainable food procurement, and market development, while microbial and insect proteins require greater emphasis on research and innovation, circular use of biomass and by-products, and appropriate regulatory frameworks. For cultivated meat, policy priorities are more strongly associated with research and development, scale-up, investment, and evidence-based regulatory assessment. Recent EU policy developments further reinforce this differentiated approach by emphasizing the diversification of protein sources, strengthening European protein production and value chains, and supporting research and innovation across emerging protein technologies [79].

It should also be acknowledged that direct comparisons across alternative protein systems remain challenging because the reviewed studies employ heterogeneous functional units, life-cycle assessment boundaries, technological assumptions, and economic scenarios. Consequently, differences reported in the literature should be interpreted as indicative trends rather than universally applicable performance rankings. Cost remains an important determinant of the future development of alternative protein technologies; however, meaningful comparisons of current and projected costs require technology-specific techno-economic assumptions that are not directly comparable across the four production systems. Therefore, the present review therefore considers cost as one component of the broader comparative assessment rather than providing a dedicated cost forecast.

## 7. Conclusions

This review provides an integrated assessment of four major alternative protein platforms, plant-based proteins, microbial proteins, cultivated meat, and insect proteins, by combining bibliometric mapping with a comparative analysis of their environmental, economic, technological, nutritional, and social characteristics. The bibliometric evidence demonstrates an evolution of the research landscape from technology-oriented studies toward integrated sustainability frameworks, with increasing attention to circular bioeconomy, life-cycle assessment, consumer acceptance, and protein transition. This shift suggests that future research is progressively moving beyond the evaluation of individual protein sources toward understanding their role within interconnected and resilient food systems.

The qualitative synthesis highlights that each protein category follows a distinct technological trajectory and occupies a different position within the emerging protein landscape. Plant-based proteins currently represent the most technologically mature and commercially competitive solution, benefiting from established production systems and relatively high consumer acceptance. Microbial proteins offer exceptional resource efficiency and considerable opportunities for circular bioeconomy through the utilization of alternative carbon sources and industrial side streams. Cultivated meat provides the closest biological equivalent to conventional meat and has the potential to satisfy demand for products with authentic sensory characteristics, although large-scale commercialization remains constrained by technological complexity, high production costs, and energy requirements. Insect proteins demonstrate outstanding potential for nutrient recycling and waste valorization, but their broader adoption continues to be limited primarily by consumer acceptance and regulatory challenges.

The comparative analysis further demonstrates that sustainability performance cannot be evaluated using a single indicator. Environmental efficiency, economic feasibility, technological readiness, nutritional quality, and social acceptance frequently involve trade-offs, preventing any individual protein technology from outperforming the others across all dimensions. Instead, the reviewed evidence indicates that the comparative advantages of each system arise from their different production pathways, resource requirements, and intended market applications.

This review demonstrates that no single alternative protein technology simultaneously maximizes environmental, economic, nutritional, and social performance. Instead, the future protein transition is likely to evolve toward an integrated portfolio of complementary production systems, where plant-based proteins provide immediate large-scale substitution, microbial proteins enhance resource efficiency and circularity, insect proteins facilitate nutrient recycling, and cultivated meat addresses demand for conventional meat equivalents. Consequently, future food systems should be designed around technological complementarity rather than competition among alternative protein platforms.

Future research should increasingly move beyond isolated assessments of individual protein technologies toward integrated evaluations that combine life-cycle assessment, techno-economic analysis, nutritional functionality, consumer behavior, and regulatory readiness within harmonized methodological frameworks. Such multidisciplinary approaches will provide a more robust basis for supporting evidence-based policy decisions and guiding investments in sustainable protein systems.

## Figures and Tables

**Table 1:** Keyword-based thematic clustering of alternative protein research: results from VOSviewer co-occurrence mapping.

Cluster	Keywords	Cluster Characteristics
Cluster 1 (red) *insect protein + microbial protein*	Circular economy, circular bioeconomy, environmental sustainability, recourse recovery, aquaculture, fishmeal replacement, feed	Cluster 1 reflects a production-oriented sustainability stream focused on integrating insect, microbial, and microalgae proteins into circular bioeconomy and aquaculture systems. The literature within this cluster predominantly addresses feed applications, fishmeal substitution, and resource recovery strategies that enhance resource efficiency and reduce environmental impacts.
Cluster 2 (green) *plant-based protein*	Fermentation, protein digestibility, functional properties, food waste, rheology, solubility, pea protein, legumes	Cluster 2 reflects a food technology and product development stream centered on plant-based proteins and their optimization for food applications. The literature within this cluster primarily focuses on fermentation processes, digestibility, rheological and functional properties, as well as the utilization of legumes and pea proteins to improve the quality and functionality of alternative protein products.
Cluster 3 (blue) *protein transition*	Carbon footprint, environmental impact, sustainable diets, consumer behaviour, meat alternatives (analogues, substitutes)	Cluster 3 reflects a research stream focused on consumption and the environmental implications of the transition toward alternative proteins. The literature within this cluster predominantly addresses carbon footprint reduction, sustainable diets, consumer behaviour, and the role of meat alternatives and substitutes in facilitating dietary transition.
Cluster 4 (yellow) *cultivated meat/cell-based meat*	Alternative protein, life cycle assessment, consumer acceptance, consumer perception, cellular agriculture, sustainable consumption, food neophobia	Cluster 4 reflects an emerging research stream focused on cultivated meat and cellular agriculture as innovative approaches to sustainable protein production. The literature within this cluster mainly explores life cycle assessment, consumer acceptance and perception, sustainable consumption, and psychological barriers such as food neophobia associated with novel food technologies.
Cluster 5 (purple) *sustainability*	Food security, food system, nutrition, climate change	Cluster 5 reflects a broader systemic sustainability stream linking alternative proteins to global food security and sustainable food system transformation. The literature within this cluster primarily addresses the interconnections between nutrition, climate change mitigation, and the long-term environmental performance of food systems.

**Table 2:** Comprehensive comparison of plant-based protein sources [3,4,5,8,10,11,12,13,14,15].

Protein Source	Protein (Raw, g/100 g)	Emulsification	Gelation/Structuring	WHC/OHC	Foaming	Final Products	Limitations
Legumes							
Soy	35–56	High	High	High	High	Burgers, nuggets, sausages, mince, soy milk, tofu	Allergenicity, GMO concerns, beany flavor
Pea	23–35	High	Medium	High	Medium	Chicken and fish analogues, burgers, binders	Beany flavor, moderate gel strength
Chickpea	12–31	High	Medium	High	Medium	Nuggets, meat extenders, spreads	Antinutrients
Lentil	24–30	Medium	Medium	Medium	NR	Meat extenders, burgers, nuggets	Low sulfur amino acids
Faba Bean	20–41	High	High	High	Medium	Whole-cut analogues, stabilizers, protein enrichment	Starch interactions, antinutrients
Lupin	30–46	High	High	High	Medium	Steaks, high-protein bread additives, fish analogues	Alkaloids, allergenicity
Mung Bean	14.6–32.8	High	High	High	High	Egg substitutes, sausages, viscous foods (soups)	Antinutrients
Jack Bean	23.8–40.7	Medium	NR	Medium	NR	Plant-based chicken sausage, livestock feed	Antinutrients
Bambara Nut	18.2–31.2	High	NR	Medium	High	Noodles, bread, nutritional supplements	Processing difficulty
Oilseeds							
Pumpkin Seed	25–56	Medium	Medium	High	Medium	Meat analogues	Low solubility (acidic pH)
Flaxseed	20–30	High	Medium	High	Medium	Meat emulsions, nutritional additives	Cyanogenic compounds
Chia	18–30	High	Medium	High	NR	High-moisture burgers	Fiber interference
Hemp	20–26	High	High	High	High	High-moisture analogues, snacks	Lower solubility
Peanut	22–38	High	Medium	Medium	High	Nuggets, spreads	Allergenicity
Nuts							
Almond	17–24	High	NR	Medium	Medium	Plant-based dairy, meat extenders, snacks	Allergenicity
Hazelnut	10.2–22.1	Medium	NR	Medium	NR	Confectionery, protein-enriched snacks	Allergenicity
Walnut	13.6–18.1	Medium	NR	Medium	Medium	Meat extenders, nutritional supplements	High fat content, susceptible to oxidation
Cashew	18–25	High	Medium	Medium	NR	Spreads, meat analogues	Allergenicity
Cereals & pseudo-cereals							
Wheat (Gluten)	75–80 (concentrate)	NR	High	High	NR	Seitan	Gluten intolerance
Oat	11–17	Medium	NR	High	NR	Milk alternatives, snacks	Low protein quality
Rice	7–16.4	NR	NR	Medium	NR	Baby food, gluten-free pasta	Low functionality
Quinoa	13–14	Medium	Medium	Medium	NR	Nuggets, mortadella, burgers	Saponins
Barley	9–12.5	NR	Medium	High	NR	Weaning foods, meat-like dough	Low protein
Sorghum	8–12	NR	Medium	Medium	NR	Snacks	Low solubility
Tubers							
Potato	1–2	Medium	NR	High	NR	Binders, sausages	Very low protein content

Note: The information presented in the table is synthesized from multiple bibliographic sources [3,4,5,8,10,11,12,13,14,15]. As individual protein characteristics, functional properties, applications, and limitations may be reported across several sources, the references are provided collectively rather than assigned to individual cells. NR—Not reported.

**Table 3:** Comparison of plant protein structuring technologies: microstructure outcomes, scalability, and challenges.

Structuring Technology	Structuring Approach	Microstructure Outcome	Scalability	Challenges	References
Low-moisture extrusion	Top-down	Heterogeneous expanded fibrous matrix	High	Limited control of fine fibrillar structure	Sha & Xiong [5], Tziva et al. [16], Jang & Lee [3]
High-moisture extrusion	Top-down	Aligned fibrous, layered anisotropic structure	High	High energy demand; equipment cost and maintenance	Sha & Xiong [5], Tziva et al. [16]
Shear cell technology	Predominantly Top-down	Well-defined anisotropic fibrous structure	Medium	Batch processing; limited throughput	Ulhas et al. [9], Kyriakopoulou et al. [15], Sher et al. [11]
3D printing	Bottom-up	Spatially controlled structured matrices	Low	Slow production rate; formulation sensitivity	Ulhas et al. [9], Sha & Xiong [5], Jang & Lee [3]
Electrospinning	Bottom-up	Nanofibrous protein structures	Low	Solvent use; safety and residue concerns	Ulhas et al. [9], Kyriakopoulou et al. [15]
Freeze structuring	Predominantly Top-down	Porous aligned fibrous networks via ice templating	Low	Time-consuming; ice-crystal damage control	Yuliarti et al. [17], Joshi et al. [8], Jang & Lee [3]
Wet spinning	Bottom-up	Continuous aligned protein fiber formation	Medium	High cost; complex coagulation chemistry	Ulhas et al. [9], Sher et al. [11], Kyriakopoulou et al. [15]

**Table 4:** Comparative characteristics of microbial platforms used for microbial protein production.

Microbial Platforms	Microalgae	Filamentous Fungi	Bacteria	Yeasts
Characteristics for Comparison				
Protein content [18,23]	Typically above 40% of dry weigh with a high content of essential amino acids, omega-3 fatty acids.	Approximately 30–45% of dry weight; rich in amino acids, B vitamins	50–80% of dry weight.	Approximately 30–50% of dry weight, rich in B vitamins.
Substrate for cultivation [18,23,32,34]	Wastewater, agricultural waste, CO_2_, photosynthesis in open ponds or bioreactors.	Lignocellulosic materials, food waste, sugar, molasses	Methane, methanol, hydrocarbons, food waste, industrial wastewater.	Whey waste, cellulose hydrolysates, agricultural waste
Growing methods [18,23,34]	Oxidation ponds, photobioreactors.	Immersion fermentation, solid-state fermentation.	Bioreactors, fermenters with additional post-harvest treatment.	Fermentation on cheap substrates.
Areas of application [18,23,32,34]	Food additives, animal feed, medical supplements.	Alternatives to meat, feed, food additives.	Animal feed, food additives, biotechnological developments.	Food additives, traditional products, feed ingredients.
Economic feasibility [18,23,34]	Low raw material cost, complex processing for food.	Moderate cost depending on the substrate, the risk of toxin formation increases costs.	Expensive nucleic acid removal processes, capital costs for fermenters and reactors.	Low production cost, high fermentation efficiency.

Note: The information presented in the table is synthesized from multiple bibliographic sources [18,23,32,34]. References are provided at the level of the respective comparison characteristics because individual characteristics are supported by multiple sources.

**Table 5:** Comparative characterization of cell sources for cultivated meat.

Cell Source/Cell Line	Biological Origin	Meat Tissue Formation Capacity	Main Advantages	Main Limitations	Scalability Potential	Key References
Muscle satellite cells (MuSCs)	Adult skeletal muscle tissue	High specificity for skeletal muscle fiber formation	Natural muscle identity; genetic stability; physiologically relevant differentiation	Limited expansion capacity; invasive sourcing; adherent growth requirements	Low-Medium (requires microcarriers/support matrices)	Chen et al. [35], Domagała et al. [40], Wang et al. [36], Ching et al. [39], Garbin et al. [41].
Mesenchymal stem cells (MSCs)	Bone marrow, adipose tissue, perinatal tissues	Dual contribution to muscle-like and adipose-like tissues	Accessible sources; dual tissue contribution; supportive paracrine activity	High donor-to-donor variability; reduced stability during long-term culture; limited phenotypic consistency during expansion	Medium (requires scaffold/adherent systems)	David et al. [42], Domagała et al. [40], Ching et al. [39]
Embryonic stem cells (ESCs)	Inner cell mass of blastocyst	High potential for multi-tissue meat constructs (muscle, fat, connective tissue)	Unlimited self-renewal; broad differentiation spectrum; no need for repeated harvesting	Ethical constraints; control of differentiation required; regulatory limitations	High (suspension-compatible under optimized conditions)	Escobar et al. [43], Chen et al. [35], Domagała et al. [40]
Induced pluripotent stem cells (iPSCs)	Reprogrammed somatic cells (e.g., fibroblasts)	High potential for multi-lineage tissue formation	Ethical advantage; scalable expansion; customizable cell lines	Genetic and epigenetic instability concerns; tumorigenicity risk; regulatory and consumer acceptance issues	High (adaptable to large-scale bioprocessing systems)	David et al. [42], Escobar et al. [43],

**Table 6:** Integrated bioprocessing pipeline for cultivated meat production.

Technological Component	Process Stage	Representative Technologies	Key Scale-Up Constraint	Industrial Readiness
Cell expansion systems [35,38,39,40]	Upstream biomass generation	Adherent and suspension-based culture systems; microcarrier-assisted expansion	High cost of growth factors and media; finite proliferative capacity in primary cells; variability in expansion kinetics	Medium-High (pharma-adapted; food-grade optimization required)
Bioreactor systems [35,36,40,44]	Controlled cultivation environment	Stirred-tank, perfusion, airlift, hollow-fiber bioreactors	Oxygen transfer limitations; shear stress sensitivity; heterogeneity in large-scale volumes	High (stirred-tank); Emerging (perfusion, hollow-fiber for dense tissues)
Scaffold-based structuring systems [37,41,45]	3D tissue organization	Edible plant-derived scaffolds (cellulose, soy protein), hydrogels, natural extracellular matrix analogues	Limited vascular mimicry; batch variability of natural materials; regulatory and allergen constraints	Medium-High (plant scaffolds advancing toward commercialization)
Biofabrication and assembly systems [37,38,42,45]	Spatial tissue structuring	3D bioprinting, cell sheet engineering, modular tissue assembly	Low throughput; diffusion limitations in thick tissues; need for automation and vascular-like integration	Low-Medium (pilot-scale, premium product applications)
Process intensification and control systems [35,36,41,42]	Cost reduction and efficiency optimization	Serum-free media, growth factor replacement, media recycling, AI-driven monitoring, co-culture systems	System complexity; metabolic byproduct accumulation; regulatory uncertainty of novel inputs	Emerging (key R&D focus for cost parity)

**Table 7:** Comparative profile of major insect species used for protein production.

Insect Species (Scientific Name)	Protein Content (% Dry Matter)	Primary Application Domain	Main Production Advantages	Main Limitations	Industrial Adoption Level	References
*Hermetia illucens* (Black soldier fly)	35.0–65.0%	Animal feed; Pet food; Waste valorization	Exceptional ability to upcycle organic waste (manure/catering waste); inactivates pathogens	Low water solubility; potential blackening of products upon grinding	Commercial (feed sector)	van Huis [47]; Pan et al. [48]
*Tenebrio molitor* (Yellow mealworm)	46.0–63.7%	Human food; Animal feed; Pet food; Waste valorization	Fast growth and reproduction rates; high efficiency in utilizing inorganic wastes	High fat content can limit processability; consumer neophobia in Western markets	Commercial (Authorized novel food)	Brai et al. [49]; Pan et al. [48]
*Acheta domesticus* (House cricket)	53.9–70.8%	Human food; Animal feed; Pet food	High fecundity and short lifespan; attractive nutritional profile for direct consumption	Potential for cross-reactive food allergies; concerns over pesticide residues from mass rearing	Commercial (Authorized novel food)	Skotnicka et al. [50]; Pan et al. [48]; Nachtigall et al. [51]
*Alphitobius diaperinus* (Lesser mealworm)	45.0–65.0%	Human food; Animal feed; Pet food	High protein quality; favorable techno-functional properties (e.g., gelling ability)	Least studied among primary species approved for human consumption	Commercial (Authorized novel food)	Skotnicka et al. [50]; Jankowski et al. [52]
*Locusta migratoria* (Migratory locust)	46.8–65.9%	Human food	Favorable sensory properties; rich mineral composition (Fe, Zn)	Concerns over residual pesticides from wild harvesting or large-scale rearing	Commercial (Authorized novel food)	Brai et al. [49]; Nachtigall et al. [51]; Tang et al. [53]
*Musca domestica* (Common housefly)	54.0–71.6%	Animal feed; Waste valorization	Highly effective tool for pig and poultry manure biodegradation	Substrate-dependent variability; post-harvest safety treatment required; low consumer acceptance	Commercial (Primarily feed)	van Huis [47]; Tang et al. [53]
*Bombyx mori* (Silkworm)	48.7–61.2%	Human food (limited); Animal feed	Established circularity as a by-product of the silk industry; high historical use	Dependence on mulberry feed; limited substrate flexibility; low standardization for food-grade production	Commercial	Pan et al. [48]; Skotnicka et al. [50]

## Data Availability

The bibliometric data used in this study were retrieved from the Scopus database. The processed data supporting the findings are available from the corresponding author upon reasonable request.
